# Supplementary material for: Probing TDP-43 condensation using an in silico designed aptamer
Source: Nat Commun. 2022 Jun 23;13:3306. doi: 10.1038/s41467-022-30944-x (PMC9226187; doi:10.1038/s41467-022-30944-x)
Supplement: Supplementary file 1 — Supplementary Information [file 41467_2022_30944_MOESM1_ESM.pdf]

Supplementary information

Probing TDP-43 condensation using an in silico designed aptamer

Elsa Zacco[1,#], Owen Kantelberg[2,#], Edoardo Milanetti[3,4], Alexandros Armaos[1], Francesco Paolo Panei[3], Jenna Gregory[5,6,7], Kiani Jeacock[2], David J. Clarke[2], Siddharthan Chandran[5,6,7], Giancarlo Ruocco[3,4], Stefano Gustincich[1], Mathew H. Horrocks[2,*], Annalisa Pastore[8,*] and Gian Gaetano Tartaglia[1,9,10,11,*]

[1] Centre for Human Technologies (CHT), Istituto Italiano di Tecnologia (IIT), Via Enrico Melen, 83, 16152 Genova , Italy;

[2] EaStCHEM School of Chemistry, University of Edinburgh, Edinburgh EH9 3FJ, United Kingdom;

[3] Department of Physics, Sapienza University, Piazzale Aldo Moro 5, 00185 Rome, Italy

[4] Center for Life Nanoscience, Istituto Italiano di Tecnologia, Viale Regina Elena 291, 00161 Rome, Italy

[5] UK Dementia Research Institute at University of Edinburgh, University of Edinburgh, Edinburgh bioQuarter, Chancellor's Building, 49 Little F

[6] Centre for Clinical Brain Sciences, University of Edinburgh, Edinburgh, UK

[7] Euan MacDonald Centre for MND Research, University of Edinburgh, Edinburgh, UK

[8] UK Dementia Research Institute at the Maurice Wohl Institute of King's College London, London SE5 9RT, UK

[9] Centre for Genomic Regulation (CRG), Dr. Aiguader 88, 08003, Barcelona, Spain;

[10] Catalan Institution for Research and Advanced Studies, ICREA, Passeig Lluís Companys 23 08010 Barcelona, Spain;

[11] Department of Biology 'Charles Darwin', Sapienza University of Rome, P.le A. Moro 5, Rome 00185, Italy

[#]Equal contribution

[*]correspondence to: mathew.horrocks@ed.ac.uk; annalisa.pastore@crick.ac.uk; gian.tartaglia@iit.it

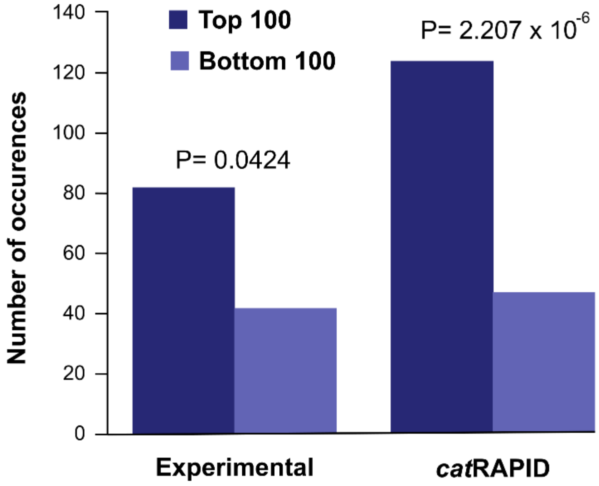

Supplementary Figure S1. We found a significant increase in the number of GU repeats in experimental CLIP-seq data (p-value 4×10^{-2}, Wilcoxon signed-ranked test, one sided). Stronger enrichment was found when ordering transcriptomic regions according to the *cat*RAPID score (p-value 2×10^{-6}, Wilcoxon signed-ranked test, one sided), thus suggesting enrichment of potential TDP-43 targets. Source data are provided as a Source Data file.

AAGUGAUGAA	CAGAAGUGAU	CGGCCACUGA	GCAGAGAGGG	GGCGGCCACG	UAUCGAGGCG
AAUGUGCACC	CAGAGAGGGU	CGGCGCUCAG	GCAGCCAUCC	GGCGGCGGCC	UCAGAAGUGA
AAUUGAUCAG	CAGAUAGACG	CGGCGGCCAC	GCAGCGAGGC	GGCUGGGGUG	UCAGAUAGAC
AAUUGAUCAG	CAGAUAGACG	CGGGCUUGUC	GCACCCUUGG	GGCUUGUCCC	UCAGAUAGAC
ACCCUUGGGU	CAGCCAUCCA	CGGGCUUGUC	GCAGAGAGGG	GGCGCUCAGA	UCCCCGAGGU
ACGAGGCCGG	CAGCGAGGCC	CGGGGCCCGG	GCAGCCAUCC	GGCGGAUCGG	UCCCCGGCCA
ACGAGGCCGG	CAUCCAGCUG	CGGUGUUGCU	GCAGCGAGGC	GGCGGCCACG	UCCCCGGCCA
ACGCUGUGGU	CCACGCUGUG	CUCAGAAGUG	GCCACGCUGU	GGCGGCGGCC	UCCCGCUGAG
ACUGAUUAUC	CCACUGAUUA	CUGAGGCUGG	GCCACUGAUU	GGCUGGGGUG	UCCCGGAGCU
AGAAGUGAUG	CCAUCCAGCU	CUGAUUAUCG	GCCAUCCAGC	GGCUUGUCCC	UCGAGGCGAU
AGACGAGGCC	CCCCGAGGUC	CUGCAGAGAG	GCCCCUUAGG	GGCUUGUCCC	UCGGUGUUGC
AGACGAGGCC	CCCCGGCCAC	CUGCAGCCAU	GCCCGGCGCU	GGGAGUGAAU	UGAAUGUGCA
AGAGAGGGUG	CCCCGGCCAC	CUGCGGGGCC	GCCCUGCGGG	GGGCCCGGCG	UGAAUUGAUC
AGAGGGUGGG	CCCCUUAGGC	CUGGCCCUGC	GCCGGGCUUG	GGGCCUGCAG	UGAAUUGAUC
AGAUAGACGA	CCCGAGGUCC	CUGGGGUGGG	GCCGGGCUUG	GGGCGGAUCG	UGAGGCUGGG
AGAUAGACGA	CCCGCUGAGG	CUGUGGUCCC	GCCUGCAGAG	GGGCUUGUCC	UGAUCAGAUA
AGCCAUCCAG	CCCGGAGCUG	CUUAGGCGGC	GCCUGCAGCC	GGGCUUGUCC	UGAUCAGAUA
AGCGAGGCCC	CCCGGCCACU	CUUGCCUGCA	GCGAGGCCCC	GGGGAGUGAA	UGAUGAAUUG
AGCUGGCCCU	CCCGGCGCUC	CUUGGGUGGG	GCGAUUCUGA	GGGGCCCGGC	UGAUUAUCGA
AGGCCCCUUA	CCCUGCGGGG	CUUGUCCCCG	GCGCUCAGAA	GGGGCGGAUC	UGCACCCUUG
AGGCCGGGCU	CCCUUAGGCG	CUUGUCCCCG	GCGGAUCGGU	GGGGUGGGGC	UGCAGAGAGG
AGGCCGGGCU	CCCUUGGGUG	GAAGUGAUGA	GCGGCCACGC	GGGUGGGCCU	UGCAGCCAUC
AGGCGAUUCU	CCGAGGUCCC	GAAUGUGCAC	GCGGCGGCCA	GGGUGGGGAG	UGCCUGCAGA
AGGCGGCGGC	CCGCUGAGGC	GAAUUGAUCA	GCGGGGCCCG	GGGUGGGGCG	UGCGGGGCCC
AGGCUGGGGU	CCGGAGCUGG	GAAUUGAUCA	GCUCAGAAGU	GGUCCCCGAG	UGCUUGCCUG
AGGGUGGGGA	CCGGCCACUG	GACGAGGCCG	GCUGAGGCUG	GGUCCCGGAG	UGGCCCUGCG
AGGUCCCGGA	CCGGCGCUCA	GACGAGGCCG	GCUGGCCCUG	GGUGGGCCUG	UGGGCCUGCA
AGUGAAUGUG	CCGGGCUUGU	GAGAGGGUGG	GCUGGGGUGG	GGUGGGGAGU	UGGGGAGUGA
AGUGAUGAAU	CCGGGCUUGU	GAGCUGGCCC	GCUGUGGUCC	GGUGGGGCGG	UGGGGCGGAU
AUAGACGAGG	CCUGCAGAGA	GAGGCCCCUU	GCUUGCCUGC	GGUGUUGCUU	UGGGGUGGGG
AUAGACGAGG	CCUGCAGCCA	GAGGCCGGGC	GCUUGUCCCC	GUCCCCGAGG	UGGGUGGGCC
AUCAGAUAGA	CCUGCGGGGC	GAGGCCGGGC	GCUUGUCCCC	GUCCCCGGCC	UGGUCCCCGA
AUCAGAUAGA	CCUUAGGCGG	GAGGCGAUUC	GGAGCUGGCC	GUCCCCGGCC	UGUCCCCGGC
AUCCAGCUGA	CCUUGGGUGG	GAGGCUGGGG	GGAGUGAAUG	GUCCCGGAGC	UGUCCCCGGC
AUCGAGGCGA	CGAGGCCCCU	GAGGGUGGGG	GGAUCGGUGU	GUGAAUGUGC	UGUGCACCCU
AUCGGUGUUG	CGAGGCCGGG	GAGGUCCCGG	GGCCACGCUG	GUGAUGAAUU	UGUGGUCCCC
AUGAAUUGAU	CGAGGCCGGG	GAGUGAAUGU	GGCCACUGAU	GUGCACCCUU	UGUUGCUUGC
AUGAAUUGAU	CGAGGCGAUU	GAUAGACGAG	GGCCCCUUAG	GUGGGCCUGC	UUAGGCGGCG
AUGUGCACCC	CGAGGUCCCG	GAUAGACGAG	GGCCCGGCGC	GUGGGGAGUG	UUAUCGAGGC
AUUAUCGAGG	CGAUUCUGAU	GAUCAGAUAG	GGCCCUGCGG	GUGGGGCGGA	UUCCCGCUGA
AUUCUGAUCU	CGCUCAGAAG	GAUCAGAUAG	GGCCGGGCUU	GUGGUCCCCG	UUGAUCAGAU
AUUGAUCAGA	CGCUGAGGCU	GAUCGGUGUU	GGCCGGGCUU	GUGUUGCUUG	UUGAUCAGAU
AUUGAUCAGA	CGCUGUGGUC	GAUGAAUUGA	GGCCUGCAGC	GUUGCUUGCC	UUGCCUGCAG
CACCCUUGGG	CGGAGCUGGC	GAUUAUCGAG	GGCGAUUCUG	UAGACGAGGC	UUGCUUGCCU
CACGCUGUGG	CGGAUCGGUG	GAUUCUGAUC	GGCGCUCAGA	UAGACGAGGC	UUGGGUGGGC
CACUGAUUAU	CGGCCACGCU	GCACCCUUGG	GGCGGAUCGG	UAGGCGGCGG	UUGUCCCCGG
					UUGUCCCCGG

Supplementary Table S1. Candidate aptamers undergoing RNA and Protein Fitness scoring.

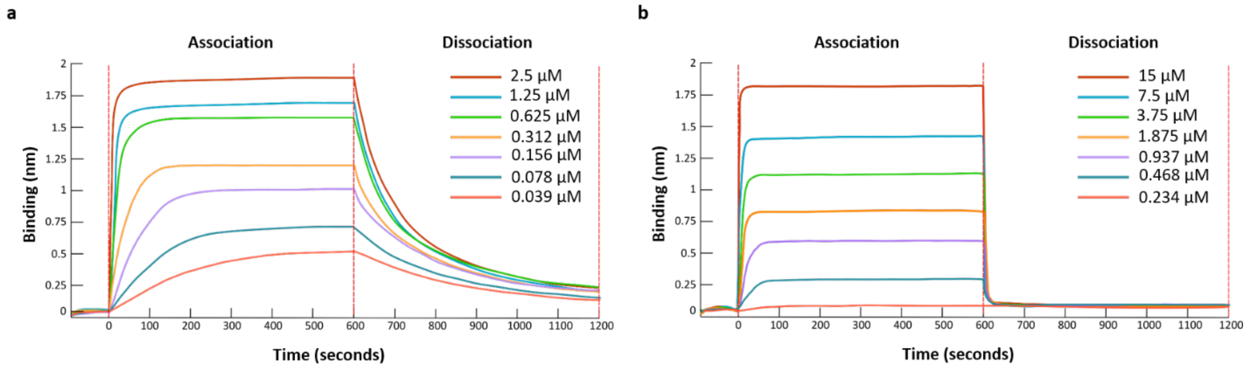

Supplementary Figure S2. **Example of binding curves of RRM1-2 with the aptamers.** The sensorgrams show the association and dissociation steps resulting from a bio-layer interferometry experiments performed with RRM1-2 and Apt-1 (**a**) or nApt-1 (**b**). Each curve indicates binding response as nm shift given by different protein concentrations, which range between 2.5 μM and 0.039 μM for Apt-1 and between 15 μM and 0.234 μM for nApt-1. Source data are provided as a Source Data file.

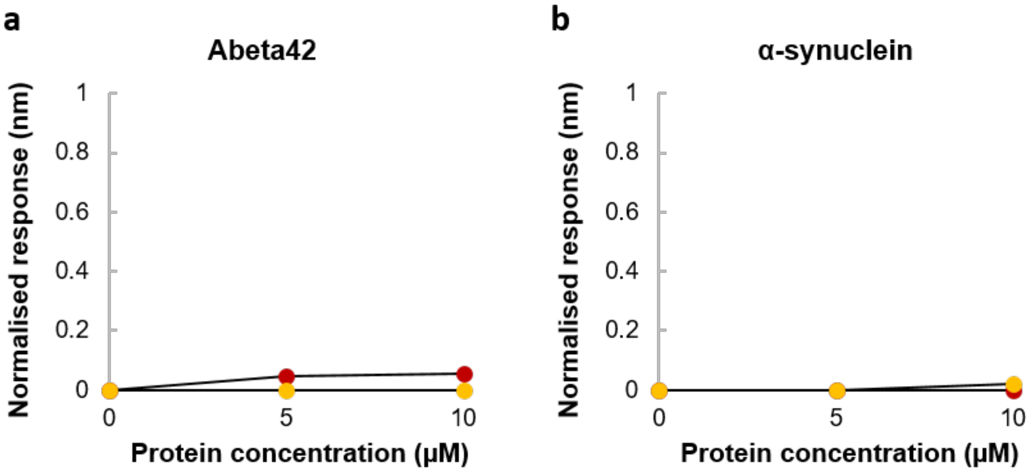

Supplementary Figure S3. Biolayer interferometry investigation of the binding between the aptamers and control amyloidogenic proteins. a) Binding curves for Abeta42; **b)** Binding curves for α-synuclein, red: Apt-1; yellow: nApt-1. At the highest tested concentration of 10 µM no binding was observed for either protein.

	Apt-1 and nApt-1 systems
Simulation time step	2 femtoseconds
Acquisition time step	100 picoseconds
algorithm of integration	Leap-frog
MD software	GROMACS
Force field	AMBER99
Total time	1 µsecond*
Reference Temperature	300K
Temperature coupling	modified Berendsen
Reference Pressure	1 bar
Pressure coupling	Parinello-Rahman
box size	1 nm distance from structure
constraint - algorithm	LINCS
RMSD fitting reference	Protein + RNA
RMSD calculation reference	Protein + RNA

	Apt-1	nApt-1
Total number of atoms	104808	72955
Number of water molecules	33902	23282

* The time is increased by steps of 1 µsecond in the case the MD simulation is not converged in at least the 60% fraction of the last microsecond.

Supplementary Table S2. Top: Parameters defined for the Molecular Dynamics of Apt-1 and nApt-1 with RRM1-2; **Bottom:** Atom counts.

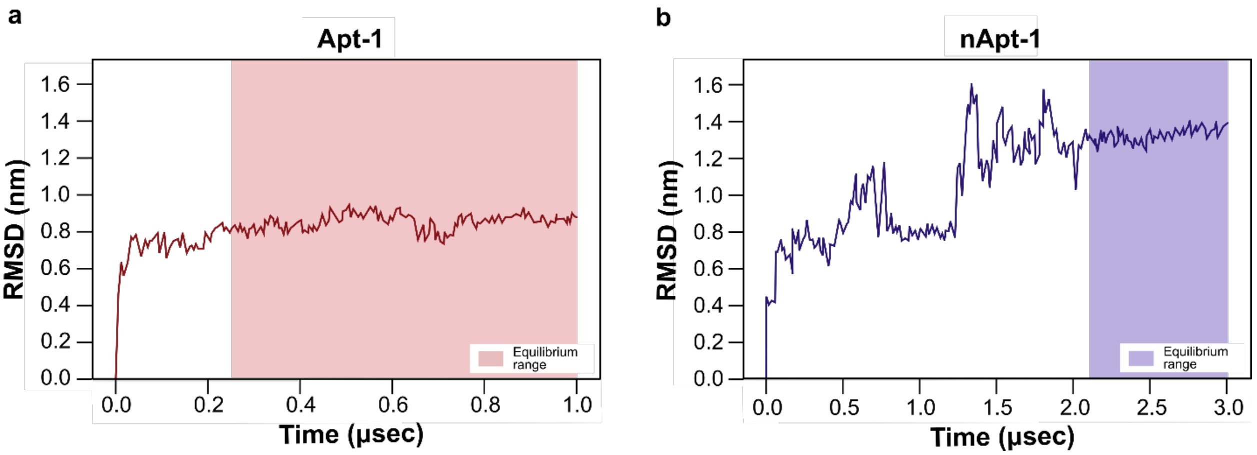

Supplementary Figure S4. MD trajectories were run in explicit solvent. Simulations were carried out setting the system temperature to 300 K. The equilibrium conditions were evaluated based on the Root Mean Square Deviation (RMSD). Starting from 1 μsec, we prolonged the simulation of 1 μsec to confirm that the RMSD had only negligible fluctuations (<1 Å) in at least the 60% of the trajectory.

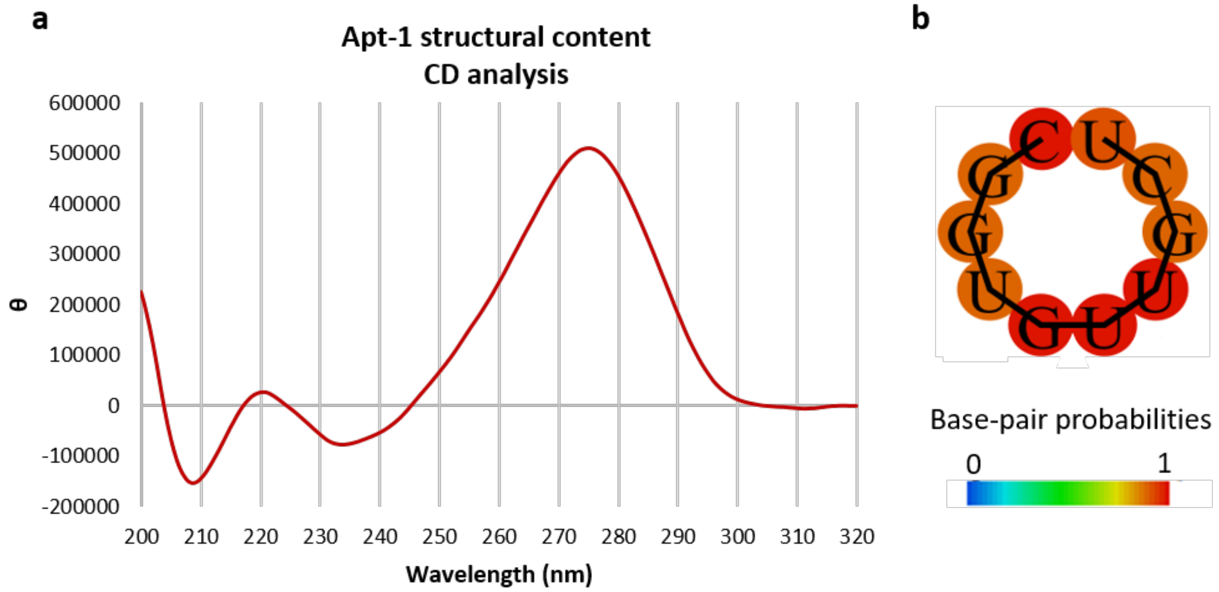

Supplementary Figure S5. Structural analysis of Apt-1. a) Circular dichroism analysis of the aptamer Apt-1, in which the spectrum displays the typical maxima and minima of an RNA sequence without any noteworthy secondary structure; **b)** Prediction of the structure of Apt-1 by means of the algorithm "RNAfold", showing very poor base-pair probabilities and emphasizing the linear nature of the sequence.

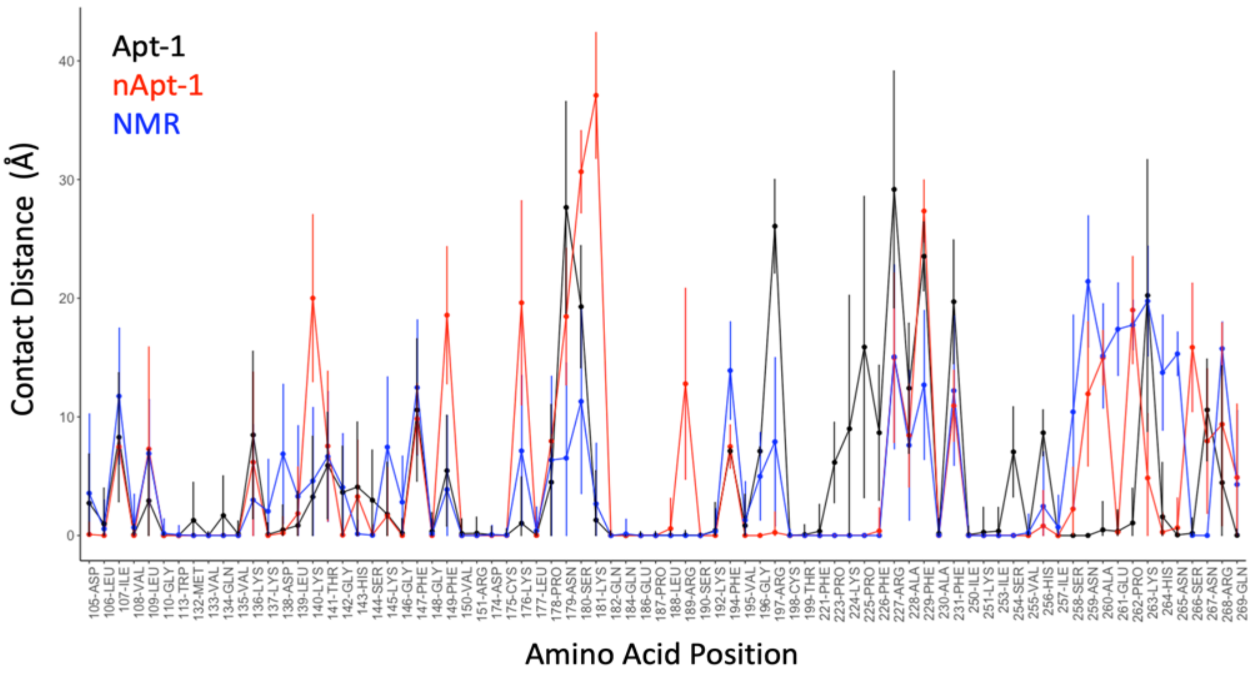

Supplementary Figure S6. Contacts of the RRM1-2 binding site with Apt-1, nApt-1 and NMR structures. Average and standard deviation of the distances are derived considering the simulation at equilibrium (Supplementary Figure S4).

a

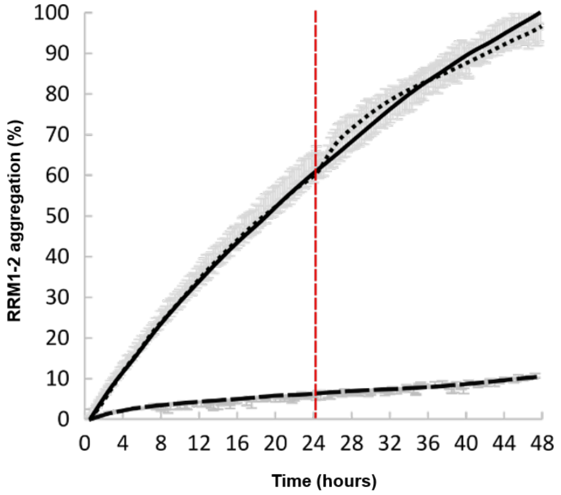

b

K_d at t_0	K_d at 24h	K_d at 48h
94 nM	110 nM	201 nM

Supplementary Figure S7. **Aggregation of RRM1-2 in the presence and absence of Apt-1 and binding affinities at different time points. a) Continuous line:** RRM1-2 by itself; **long-dashed line:** RRM1-2 with Apt-1 co-incubated with the protein since t_0; **short-dashed line:** RRM1-2 with Apt-1 added after 24 hours from the start of the assay (red vertical dashed line indicate the time of addition of Apt-1). Aggregation was measured in triplicates on a 96-well plate by means of the dye Proteostat, blanked with the buffer and normalised by considering the highest value reached after 48 hours to be 100 %. The experiment was repeated three times. **b)** K_d values reported for the pair RRM1-2—Apt-1 when adding the aptamer at t_0 (column 1), 24 hours (column 2) or 48 hours (column 3) from the start of the aggregation process of RRM1-2. The K_d was determined with BLI as reported in the "Materials and Methods" section, with the exception that RRM1-2 was left to aggregate at 37 °C under constant dual-orbital shaking. The experiment was performed in triplicate. Source data are provided as a Source Data file.

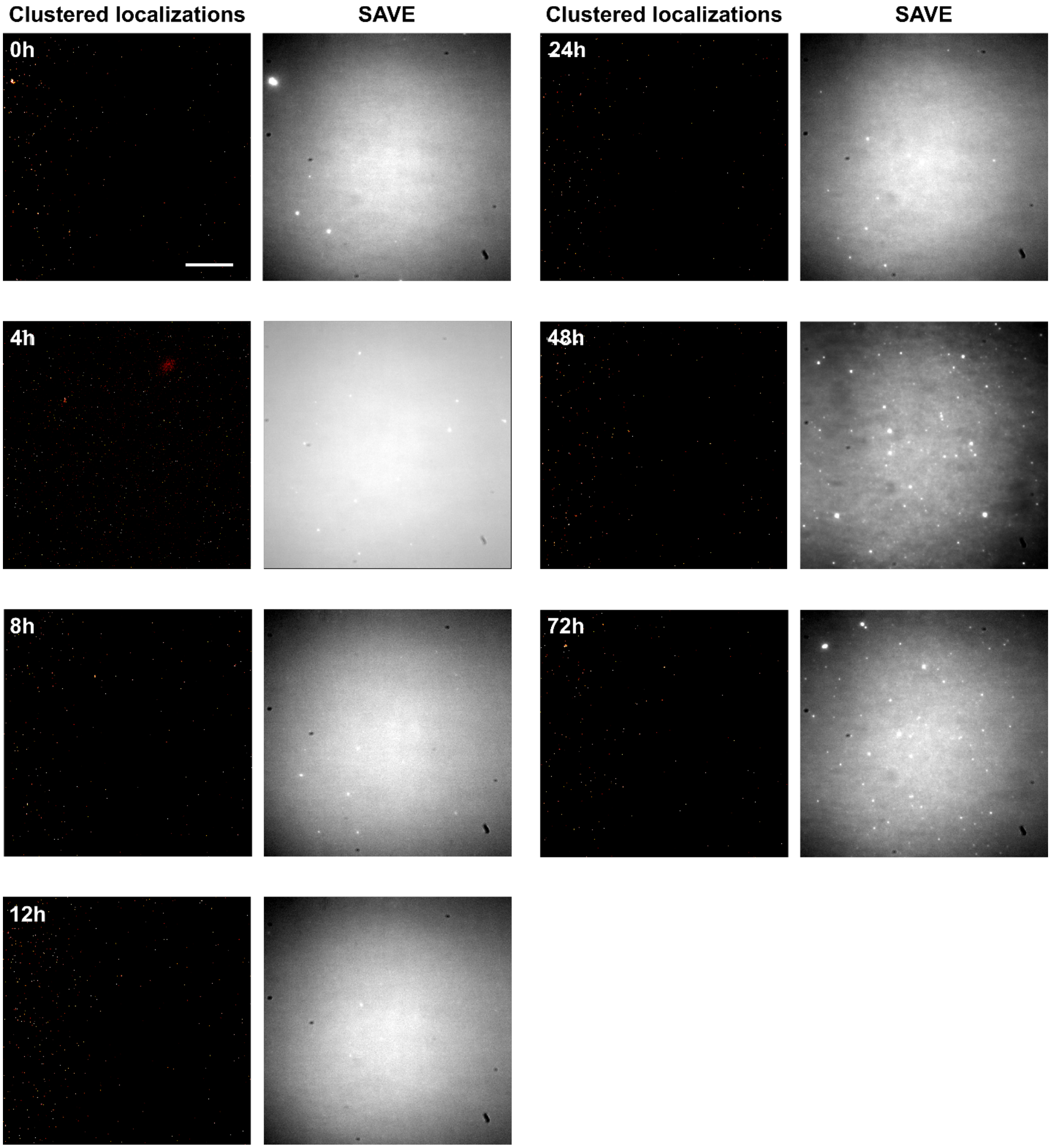

Supplementary Figure S8: Representative full fields of view at each timepoint. Clustered localizations (left panels, with time stamps) paired with their corresponding SAVE images (right panels) shown as representative images of the total of 9 fields of view captured for each imaging timepoint (0h, 4h, 8h, 12h, 24h, 48h and 72h). Data from 3 independent experiments. Scale bar 10 µm. Source data are provided as a Source Data file[1].

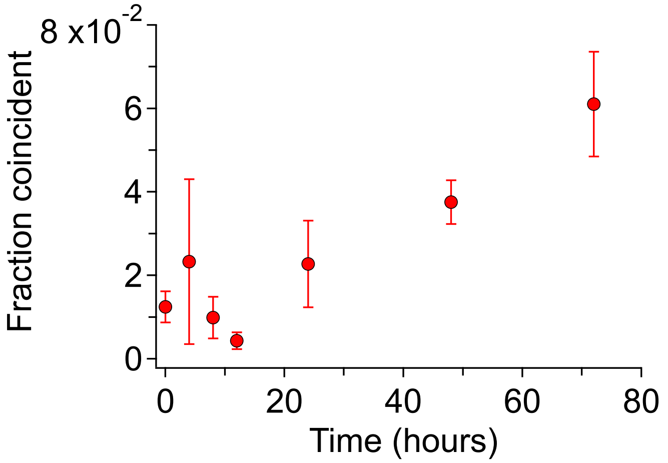

Supplementary Figure S9: Fraction of aggregates that are ThT-active. The fraction of aggregates detected using Apt1 that are ThT-active across the time course (mean +/- SD, n =3). Source data are provided as a Source Data file[1].

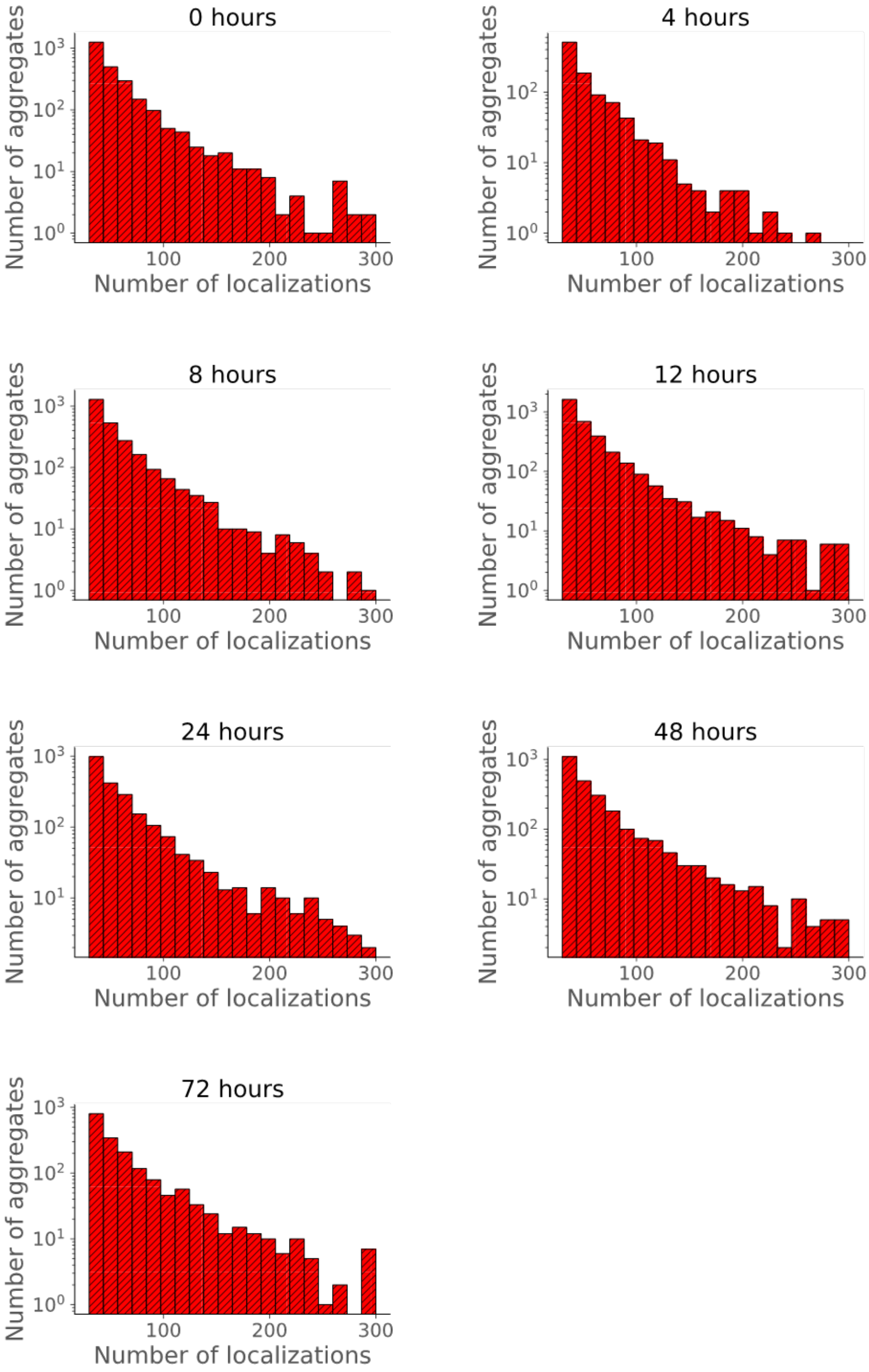

Supplementary Figure S10. Histograms of the number of localizations for each aggregate. The number of localizations measured for each of the aggregates detected (across three replica) are presented in the histograms for each time-point. Source data are provided as a Source Data file[1].

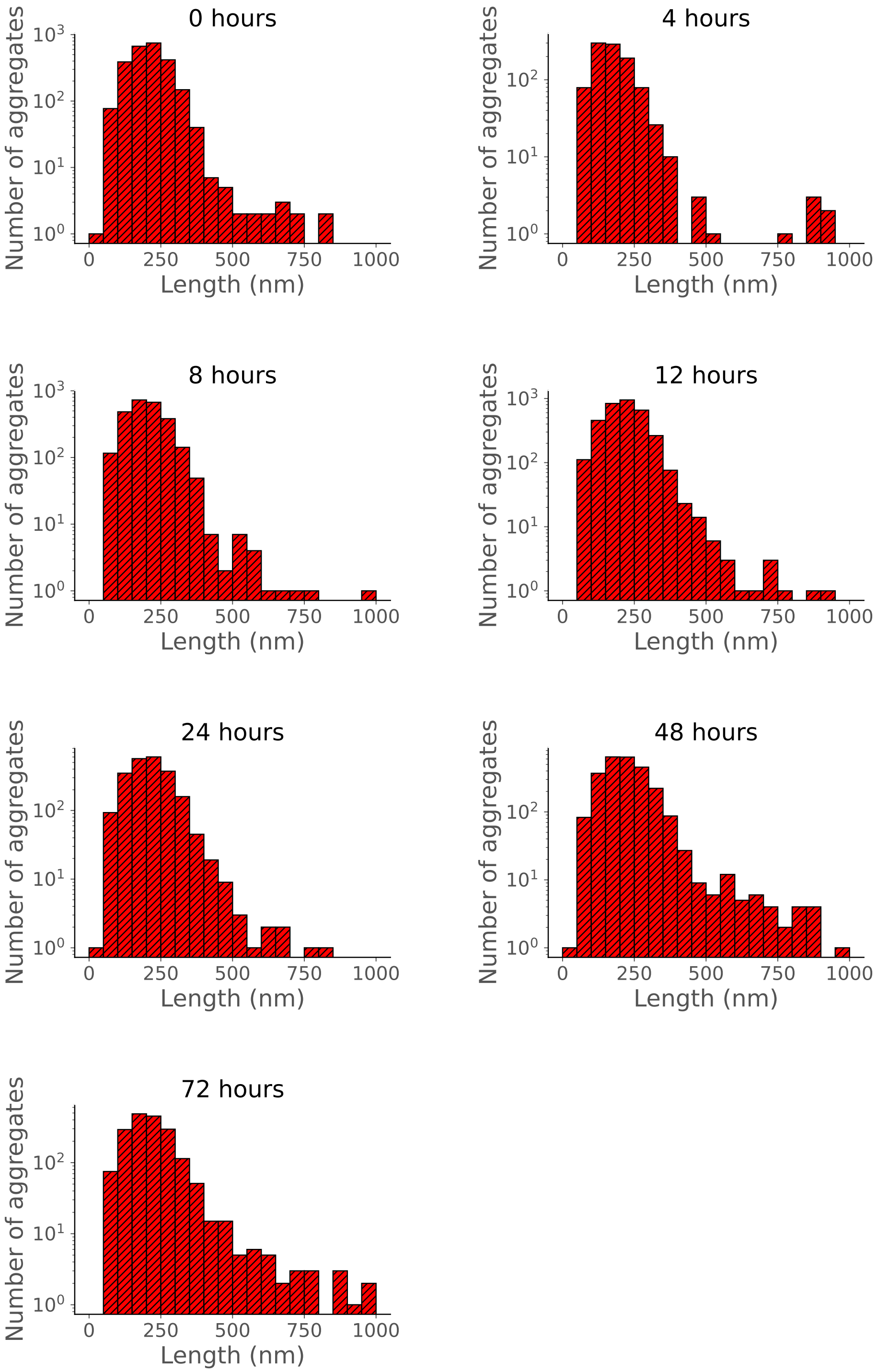

Supplementary Figure S11. Histograms of aggregate length. The lengths of all of the aggregates detected (across three replica) are presented in the histograms for each time-point. Source data are provided as a Source Data file[1].

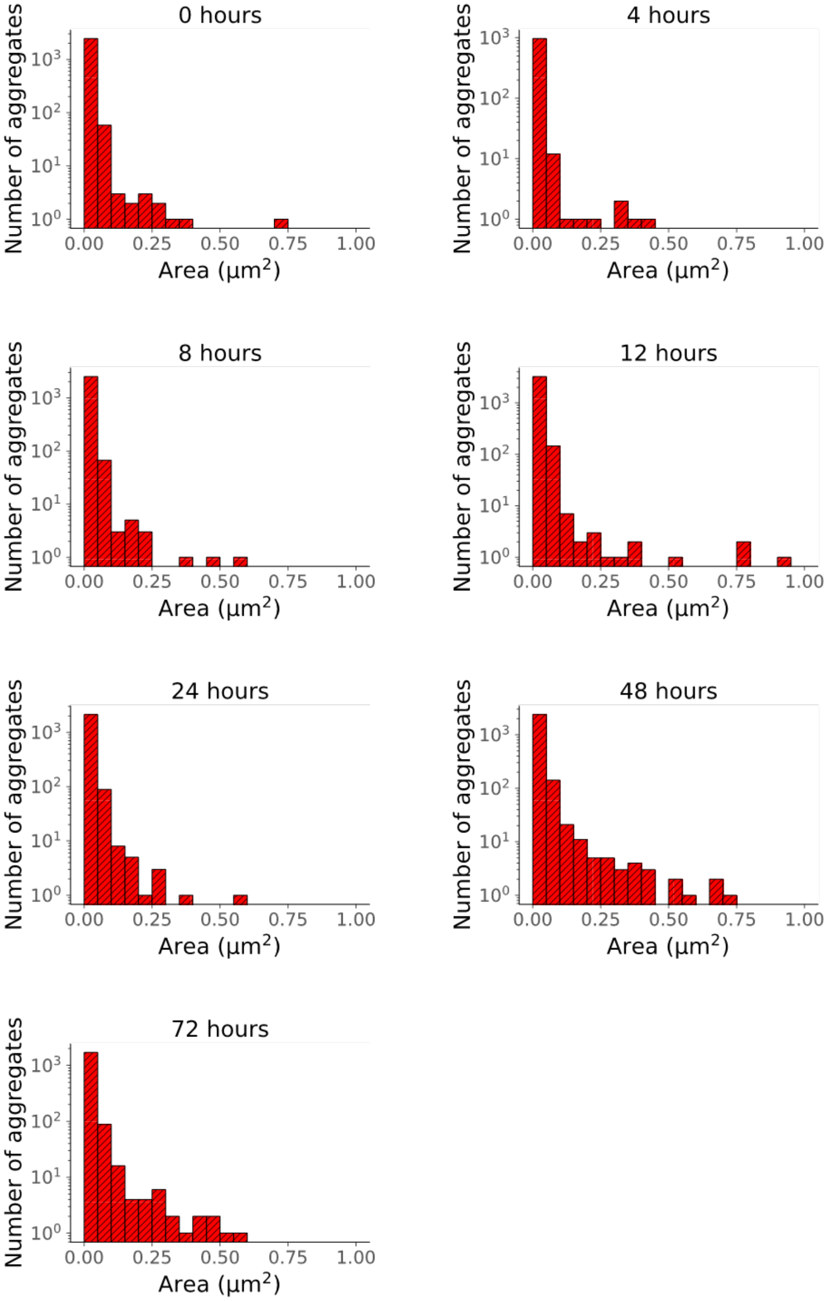

Supplementary Figure S12. **Histograms of areas for each aggregate**. The area that the localizations were projected onto for each of the aggregates detected (across three replica) are presented in the histograms for each time-point. Source data are provided as a Source Data file[1].

0 hours	4 hours	8 hours	12 hours	24 hours	48 hours	72 hours
20.58 nm	22.23 nm	20.37 nm	23.59 nm	18.56 nm	14.97 nm	17.58 nm
14.69 nm	20.48 nm	17.59 nm	24.46 nm	17.38 nm	16.11 nm	28.07 nm
14.39 nm	15.42 nm	21.34 nm	19.46 nm	24.71 nm	9.61 nm	17.57 nm
23.64 nm	15.75 nm	15.88 nm	28.05 nm	19.68 nm	21.2 nm	21.61 nm
22.39 nm	14.61 nm	17.71 nm	19.48 nm	25.11 nm	25.22 nm	24.23 nm
23.22 nm	18.73 nm	17.14 nm	19.16 nm	26.45 nm	20.23 nm	17.75 nm
17.19 nm	21.06 nm	15.88 nm	15.91 nm	19.93 nm	17.13 nm	22.04 nm
18.4 nm	14.71 nm	20.1 nm	20.34 nm	15.09 nm	16.61 nm	24.35 nm
20.02 nm	19.64 nm	18.42 nm	19.4 nm	18.89 nm	19.73 nm	23.27 nm

Supplementary Table S3: **Calculated resolutions for each image.** The resolution of each image was calculated using the Fourier Ring Correlation plug-in for imageJ, available as part of the GDSCSMLM package.

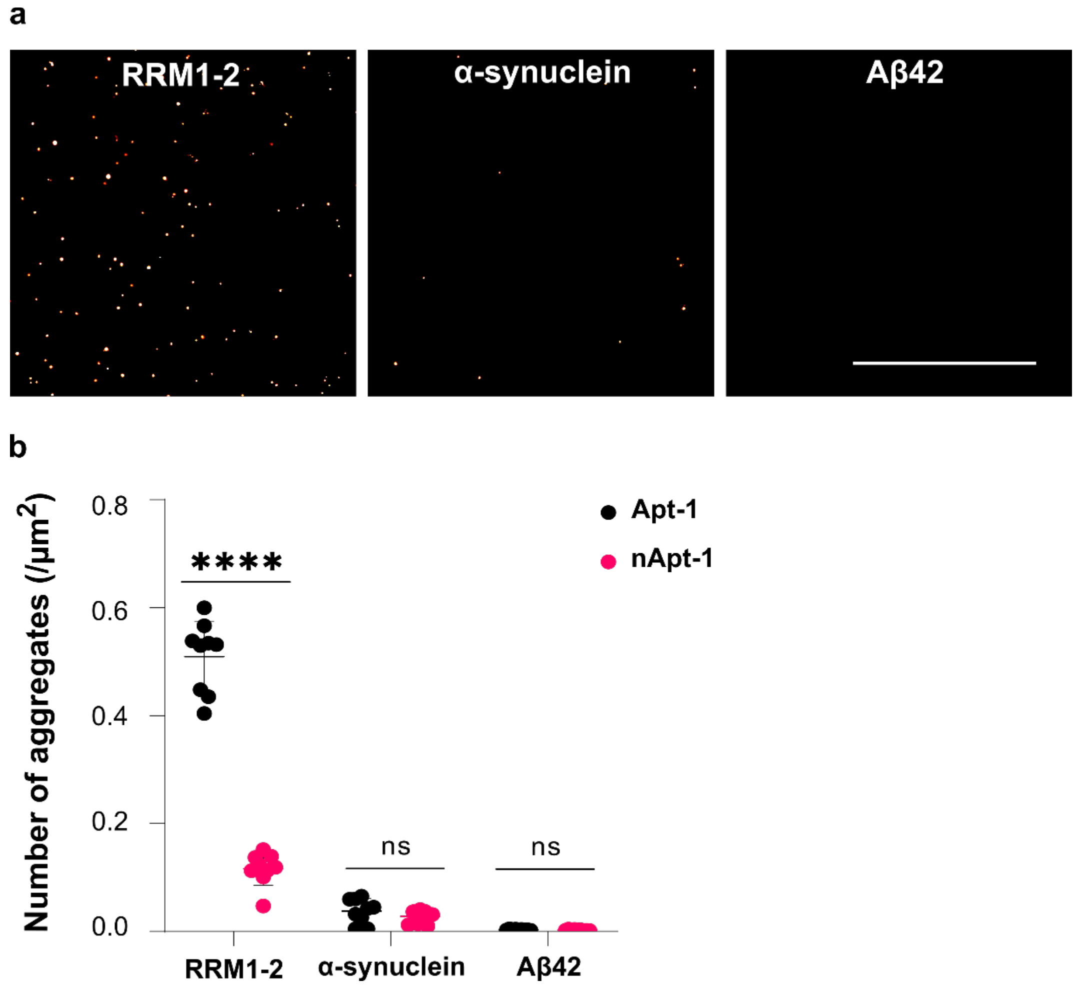

Supplementary Figure S13: **Aggregate detection of TDP-43, α-synuclein and Aβ42 with Apt-1 and nApt-1. a)** Sample fields of view of clustered aggregates imaged with Apt-1 for respective proteins. Data from 3 independent experiments. Scale bar is 5 µm. **b)** Plot of mean cluster number per μm^2 showing a significant difference in the detection of aggregates with Apt-1 and nApt-1 only for RRM1-2. The data shown are means ± SD of 9 fields of view. ****$p<0.0001$, ns $p>0.05$; analyzed by t-test. Source data are provided as a Source Data file[1].

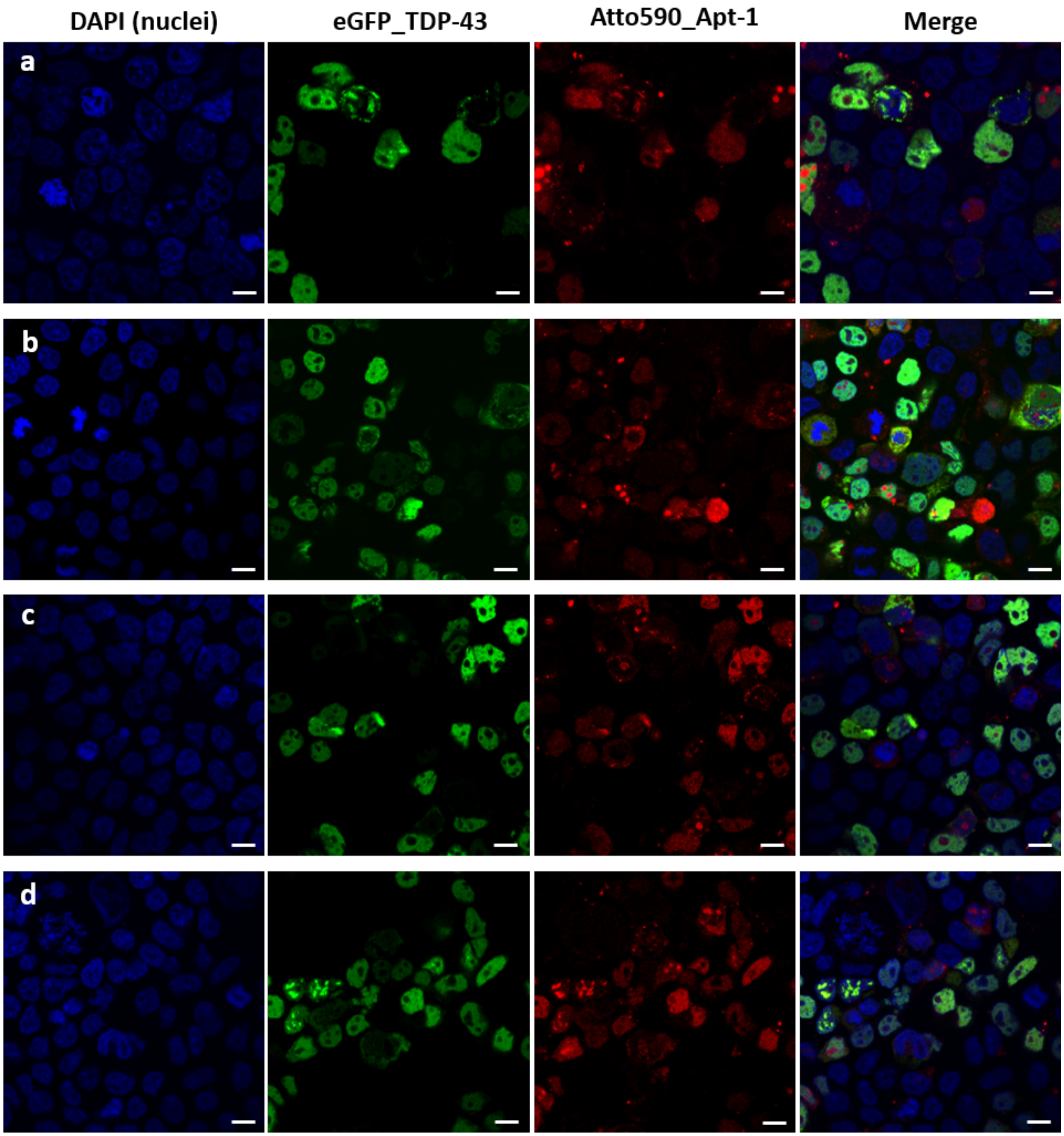

Supplementary Figure S14: **Wide field views of HeK 293T cells overexpressing TDP-43 and Apt-1. a-d)** The figures report examples of the three fluorescence channels (blue, green and red) and the merged images acquired 24 hours after the cells were co-transfected with the plasmid for the overexpression of eGFP_TDP-43 and Atto590_Apt-1. The images show similar distribution of the aptamer and its target protein within living cells. Data from three independent experiments, each time in duplicates. Scale bar = 7 µm.

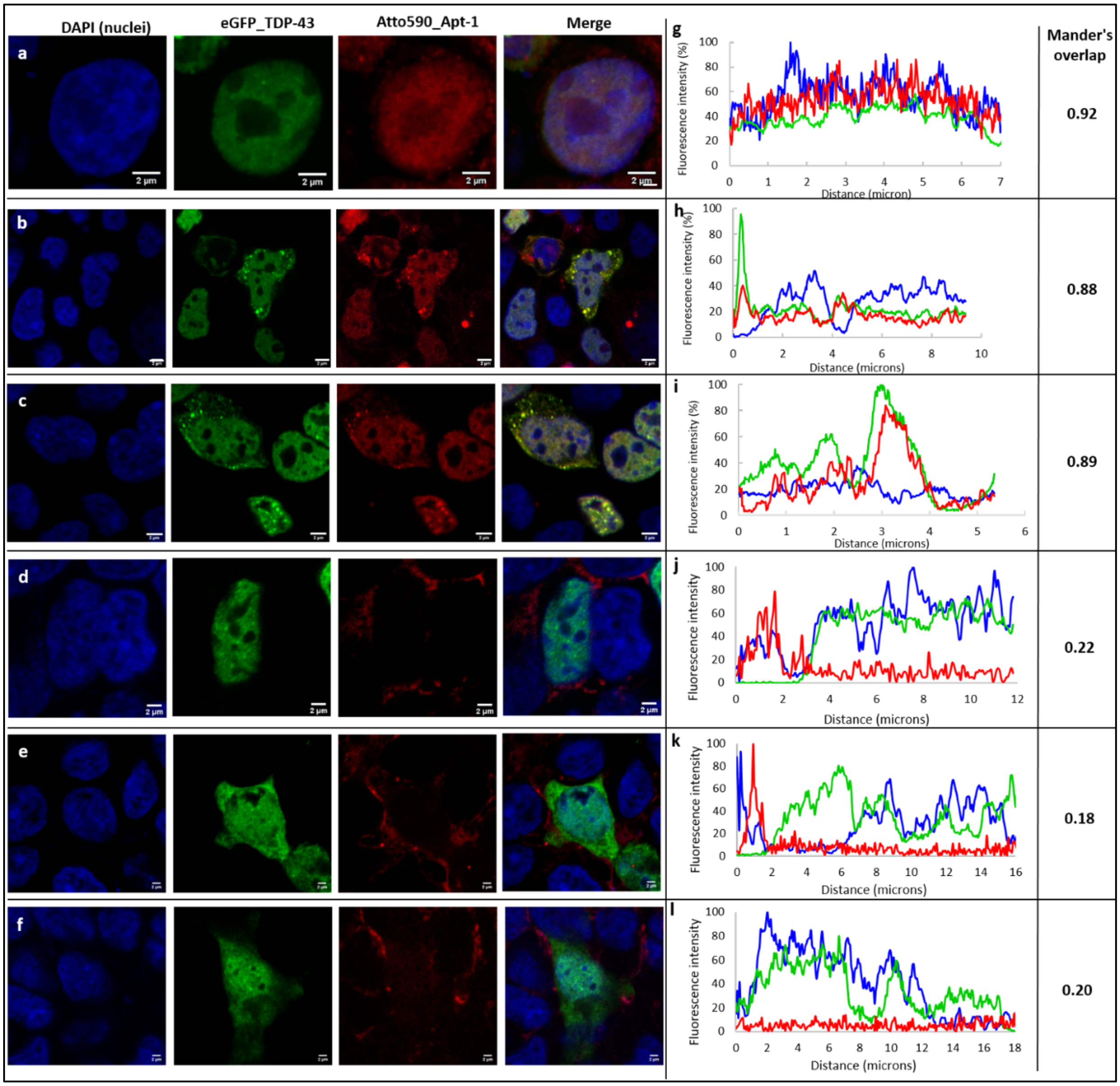

Supplementary Figure S15. Supplementary Fig. S6: Distribution of TDP-43 and Apt-1 or nApt-1 in living cells. a-c): Cells co-transfected with eGFP_TDP-43 and Atto590_Apt-1 showing overlapping distribution of the two fluorophores; **d-f):** Cells co-transfected with eGFP_TDP-43 and Atto590_nApt-1, in which the aptamer does not co-localise with the protein TDP-43; **g-i):** Fluorescence profiles of DAPI, eGFP and Atto590 showing the distribution of the green of TDP-43 and the red of Apt-1 **(g-i)** or nApt-1 **(j-i)**, across the correspondent cell on the left; Right hand-side panel: Mander's overlap values relative to the cell displayed on the left. Scale bar = 2 µm.

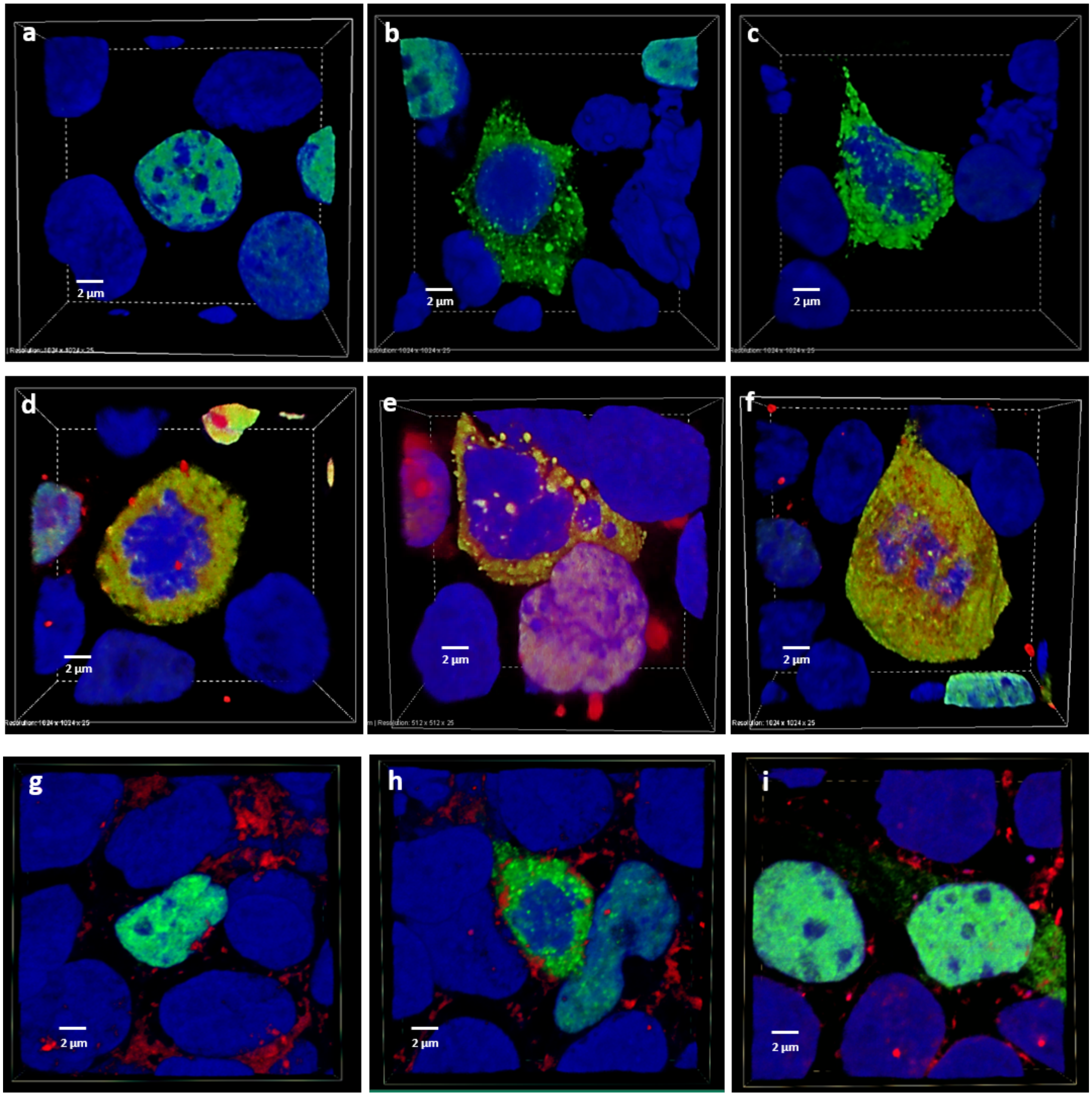

Supplementary Figure S16. Three-dimensional images of Hek 293T cells overexpressing eGFP_TDP-43. The selected examples refer to cells with nuclear TDP-43 distribution **(a)** or cytosolic aggregated TDP-43 **(b-c)**, and to cells co-transfected with Atto_Apt-1 **(d-f)** in which the aptamer overlaps with TDP-43, giving the yellow colour (derived from the green of eGFP_TDP-43 and the red of Atto_Apt-1); (**g-i**) show cells co-transfected with eGFP_TDP-43 and Atto_nApt-1, in which instead the aptamer does not recognise the target protein. 3D images average size: 20 x 20 x 12.5 µm; Z: 0.5 µm. Scale bar = 2 µm.

Aggregate imaging			
**Name**	**Sequence 5'-3'**	**Modifications**	**Supplier**
Apt-1 ATTO590	CGGUGUUGCU_ATTO590	3' ATTO-590 NHS Ester	ATDBio
Apt-1_ATTO590 2Fluoro-C	C^FGGUGUUG^{F}CU_ATTO590	"F" indicates Fluorination of the base. 3' ATTO-590 NHS Ester.	ATDBio
nApt-1_ATTO590 2Fluoro-G	ATTO590_AG^{F}CAACACCG^F	"F" indicates Fluorination of the base. 5' ATTO-590 NHS Ester.	ATDBio
**Cell imaging**			
**Name**	**Sequence 5'-3'**	**Modifications**	**Supplier**
Apt-1_ATTO590_2Fluoro-C	C^FGGUGUUG^{F}CU_ATTO590	"F" indicates Fluorination of the base. 3' ATTO-590 NHS Ester.	Integrated DNA Technologies
nApt-1_ATTO590_2Fluoro-G	ATTO590_AG^{F}CAACACCG^F	"F" indicates Fluorination of the base. 5' ATTO-590 NHS Ester.	Integrated DNA Technologies
**Biolayer interferometry**			
**Name**	**Sequence 5'-3'**	**Modifications**	**Supplier**
Apt-1_Biotin	CGGUGUUGCU-Biotin	3' Biotinylation	Sigma Aldrich
nApt-1_Biotin	Biotin-AGCAACACCG	5' Biotinylation	Sigma Aldrich

Supplementary Table S4: **Aptamer sequences and modifications.** Different modifications were used for Apt-1 and nApt-1 depending on the experimental application.

#

1. Zacco, E. *et al.* Probing TDP-43 Condensation using an In Silico Designed Aptamer. *Zenodo* (2022) doi:10.5281/zenodo.6533779.
