# Supplementary material for: Probing TDP-43 condensation using an in silico designed aptamer
Source: Nat Commun. 2022 Jun 23;13:3306. doi: 10.1038/s41467-022-30944-x (PMC9226187; doi:10.1038/s41467-022-30944-x)
Supplement: Supplementary file 3 — Reporting Summary [file 41467_2022_30944_MOESM3_ESM.pdf]

nature portfolio

Corresponding author(s): Gian Gaetano Tartaglia
Annalisa Pastore
Mathew Horrocks

Last updated by author(s): 06/05/2022

Reporting Summary

Nature Portfolio wishes to improve the reproducibility of the work that we publish. This form provides structure for consistency and transparency in reporting. For further information on Nature Portfolio policies, see our Editorial Policies and the Editorial Policy Checklist.

Statistics

For all statistical analyses, confirm that the following items are present in the figure legend, table legend, main text, or Methods section.

n/a	Confirmed	
☐	☒	The exact sample size (n) for each experimental group/condition, given as a discrete number and unit of measurement
☐	☒	A statement on whether measurements were taken from distinct samples or whether the same sample was measured repeatedly
☐	☒	The statistical test(s) used AND whether they are one- or two-sided<br>*Only common tests should be described solely by name; describe more complex techniques in the Methods section.*
☒	☐	A description of all covariates tested
☒	☐	A description of any assumptions or corrections, such as tests of normality and adjustment for multiple comparisons
☐	☒	A full description of the statistical parameters including central tendency (e.g. means) or other basic estimates (e.g. regression coefficient) AND variation (e.g. standard deviation) or associated estimates of uncertainty (e.g. confidence intervals)
☐	☒	For null hypothesis testing, the test statistic (e.g. F, t, r) with confidence intervals, effect sizes, degrees of freedom and P value noted<br>*Give P values as exact values whenever suitable.*
☒	☐	For Bayesian analysis, information on the choice of priors and Markov chain Monte Carlo settings
☒	☐	For hierarchical and complex designs, identification of the appropriate level for tests and full reporting of outcomes
☐	☒	Estimates of effect sizes (e.g. Cohen's d, Pearson's r), indicating how they were calculated

Our web collection on statistics for biologists contains articles on many of the points above.

Software and code

Policy information about availability of computer code

Data collection	No custom software was used. Predictions for the aptamer sequences were carried out by means of the software "catRAPID omics", version 2.0, available at http://s.tartaglialab.com/page/catrapid_omics2_group (the RNA Fitness of Apt-1 is at https://tinyurl.com/y3578hr3 and the Protein Fitness of Apt-1 is at https://tinyurl.com/evmybn73). Calculations for small sequences (<50 nucleic acids) are restricted in the webserver due to the filing of a patent but can be granted upon reasonable request under non-disclosure agreement.<br><br>For Molecular Dynamics simulations details are in Supplementary Table 2. The Hdock and SimRNA software were run online on their respective webpages in Summer 2018.<br><br>Biolayer interferometry data were collected by employing FORTEBIO Octet Data Acquisition software. Aggregation assays data were acquired my means of Tecan SparkControl™ software. TIRF microscope images were acquired by the Nikon Application software Ti2 Control, version 2.00 and automated using the open-source microscopy platform µManager. Confocal microscopy images were acquired with Nikon NIS-Elements Advanced Research version 5.30.02, acquisition mode.
Data analysis	For aptamer optimisation, no specific software but statistical analyses were employed as detailed in "Online methods". For the definition of the binding affinities, data were analyzed using FORTEBIO Octet® Data Analysis HT software. Aggregation assays were processed and plotted on the Tecan Magellan Data Analysis software and their statistical analysis was performed with Microsoft Excel (version 16.6). PAINT and SAVE image analysis was done using PeakFit plugin (an imageJ/Fiji plugin of the GDSC Single Molecule Light Microscopy package) and DBSCAN algorithm 57 in Python 3.8 (sklearn v0.24.2) (code available on Zenodo- 10.5281/zenodo.6533337). Confocal microscopy images were analyzed with the Nikon software NIS-Elements Advanced Research version 5.30.02, analysis mode.

For manuscripts utilizing custom algorithms or software that are central to the research but not yet described in published literature, software must be made available to editors and reviewers. We strongly encourage code deposition in a community repository (e.g. GitHub). See the Nature Portfolio guidelines for submitting code & software for further information.

Data

Policy information about availability of data

All manuscripts must include a data availability statement. This statement should provide the following information, where applicable:

- Accession codes, unique identifiers, or web links for publicly available datasets
- A description of any restrictions on data availability
- For clinical datasets or third party data, please ensure that the statement adheres to our policy

The source data for Figures 2a, 2b, 4c, 4d, 4g, 5b-g as well as Supplementary figures 1, 2, 7, 9, 10, 11, 12, 13b are provided. The super-resolution and SAVE image data generated in this study have been deposited in the Zenodo database under accession code 6533779 (https://doi.org/10.5281/zenodo.6533779). Not all calculations for aptamers are publicly available due to the filing of a patent but can be provided upon reasonable request under non-disclosure agreement.

Field-specific reporting

Please select the one below that is the best fit for your research. If you are not sure, read the appropriate sections before making your selection.

[x] Life sciences [ ] Behavioural & social sciences [ ] Ecological, evolutionary & environmental sciences

For a reference copy of the document with all sections, see nature.com/documents/nr-reporting-summary-flat.pdf

Life sciences study design

All studies must disclose on these points even when the disclosure is negative.

Sample size	1 ‰ transcriptomic regions with the highest catRAPID signal were selected considering the strong agreement with CLIP-seq data (AUC > 0.80). The size is considered appropriate given the total space of >10^6 nucleotides analysed. A total of 260 sequences were generated from the transcriptomic regions (length of 10 nt as suggested by inspection of available structural data). Mutagenesis on these regions was carried out generating 100 single/double variants per sequence, which is appropriate given their small size.
Data exclusions	All obtained results have been included in this manuscript. No data has been excluded and all attempts at replication were successful.
Replication	All data were collected at least in biological triplicates, each time including minimum 2 repetitions. Attempts at replication were successful.
Randomization	No special andomization was needed. All experiments within the same assay were performed under the same conditions (sample concentration, buffer, pH and temperature). All collected results have been normalized and processed in the same manner before data plotting.
Blinding	No blinding was needed as experiments dealt with physical measurements.

Reporting for specific materials, systems and methods

We require information from authors about some types of materials, experimental systems and methods used in many studies. Here, indicate whether each material, system or method listed is relevant to your study. If you are not sure if a list item applies to your research, read the appropriate section before selecting a response.

Materials & experimental systems

n/a	Involved in the study	
[x]	[ ]	Antibodies
[ ]	[x]	Eukaryotic cell lines
[x]	[ ]	Palaeontology and archaeology
[x]	[ ]	Animals and other organisms
[x]	[ ]	Human research participants
[x]	[ ]	Clinical data
[x]	[ ]	Dual use research of concern

Methods

n/a	Involved in the study	
[x]	[ ]	ChIP-seq
[x]	[ ]	Flow cytometry
[x]	[ ]	MRI-based neuroimaging

Eukaryotic cell lines

Policy information about cell lines

Cell line source(s)	HEK-293T (ATCC CRL-3216™)
Authentication	The cell line used was not authenticated
Mycoplasma contamination	The cell line has been tested negative for mycoplasm contamination (MycoAlertTM Mycoplasma Detection Kit, Lonza)
Commonly misidentified lines (See ICLAC register)	Not commonly misidentified lines were used in this study
